# Sinonasal Myxoma in an Infant: Observations on Its Distinctiveness and a Discussion on Potential Reclassification As Infantile Intraosseous Myxoid Desmoid Fibromatosis

**DOI:** 10.7759/cureus.65933

**Published:** 2024-08-01

**Authors:** Ryoichiro Kashiwagi, Hayato Maruguchi, Nibu Ken-ichi, Hiroto Terashi, Tadashi Nomura

**Affiliations:** 1 Department of Plastic Surgery, Kobe University Graduate School of Medicine, Kobe, JPN; 2 Department of Plastic Surgery, Maruguchi Skin Clinic, Kobe, JPN; 3 Department of Otolaryngology and Head and Neck Surgery, Kobe University Graduate School of Medicine, Kobe, JPN

**Keywords:** β-catenin, infant, pediatric, odontogenic myxoma, sinonasal myxoma

## Abstract

Myxomas, when they manifest in the paranasal sinuses and/or maxillae of infants, are classified as sinonasal myxomas (SNMs). We present a case of SNM in the maxilla of a 15-month-old infant. Following the initial surgical intervention, the patient unfortunately experienced a recurrence of the condition. However, a subsequent surgery employing marginal excision was performed, and since then, no further recurrence has been reported. SNM exhibits consistent clinical features and histological characteristics that are distinct from those of odontogenic myxomas. Furthermore, in this case, immunohistochemical staining was positive for β-catenin, whereas odontogenic myxomas are generally negative for β-catenin staining. Another study reported that SNMs share genetic mutations with desmoid tumors, which are not observed in odontogenic myxomas. This suggests that this entity is distinct from odontogenic myxomas, leading us to propose that it may indeed represent a separate disease entity. This fact may lead to the reclassification of the disease and, ultimately, to changes in treatment strategies.

## Introduction

Myxomas are slow-growing, locally aggressive, benign neoplasms derived from mesenchymal elements [1]. Sinonasal myxomas (SNMs), in particular, are extremely rare myxomas occurring in the paranasal sinuses and/or maxillae of infants [1]. Herein, we report a case of SNM located in the maxilla of a 15-month-old infant. The case findings propose that SNM could be a myxoid variant of desmoid fibromatosis, warranting a reclassification of this rare tumor.

## Case presentation

The patient, a 15-month-old female infant, was brought to medical attention when her mother noticed swelling on the left side of the nasal dorsum a week prior to the initial visit. An ophthalmologist at a different hospital was consulted. Initially, acute dacryocystitis was suspected; however, as the contents could not be aspirated by puncture and the location of the mass was atypical for dacryocystitis, the patient was referred to the plastic surgery department of that hospital under the suspicion of a tumorous lesion.

**Figure 1:**
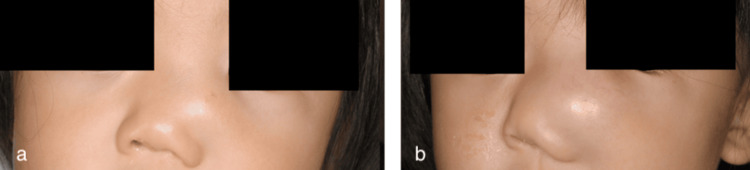
(a) Photo from the first visit to the previous hospital. The tumor was located on the left side of the nasal dorsum and measured 20 mm. (b) Three weeks after the first visit to the previous hospital, a preoperative photo of the first operation. The tumor had grown to 30 mm, displacing the lower eyelid and nasal dorsum.

**Figure 2:**
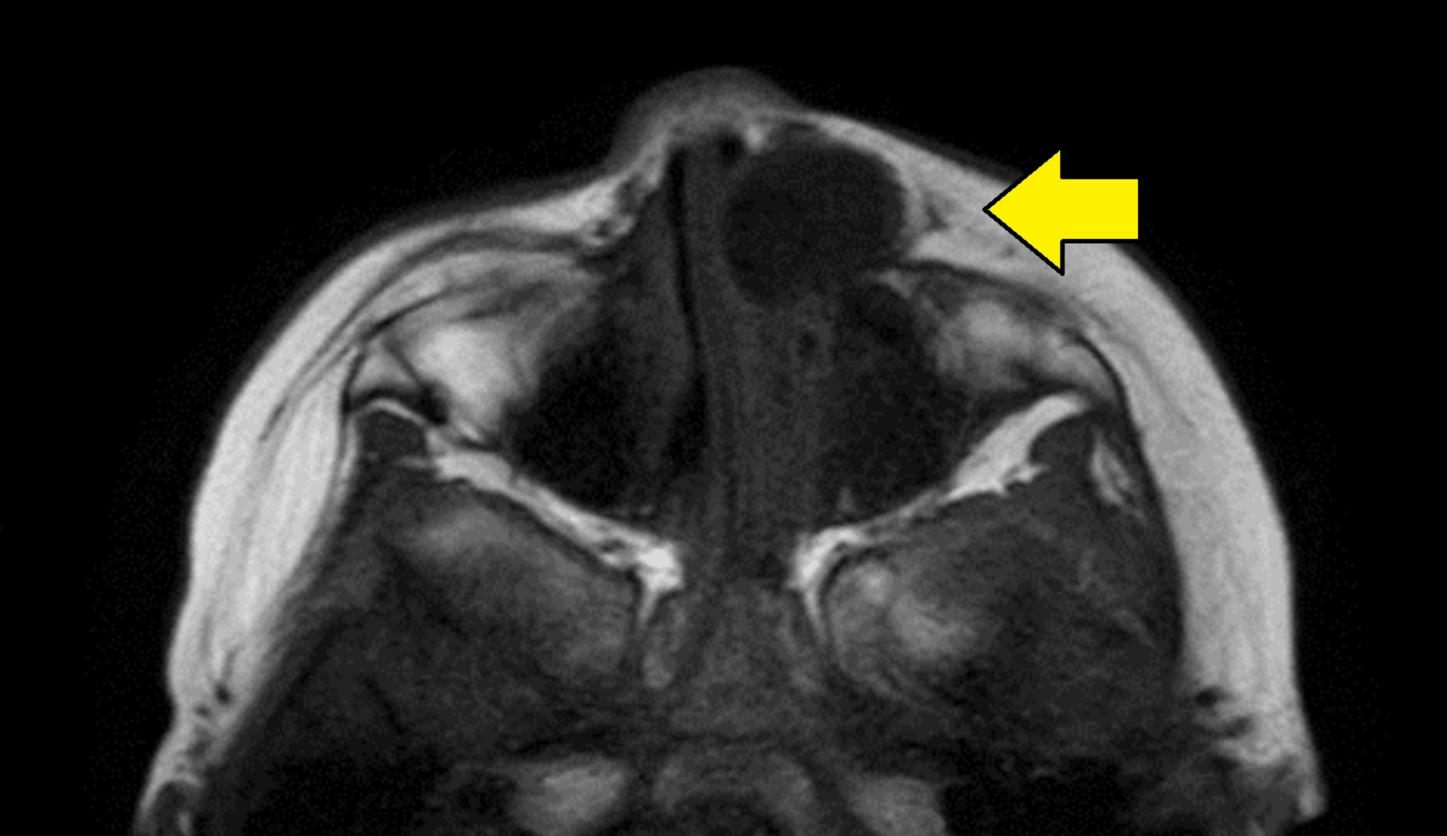
T1-weighted image showing a cystic lesion with low signal intensity.

**Figure 3:**
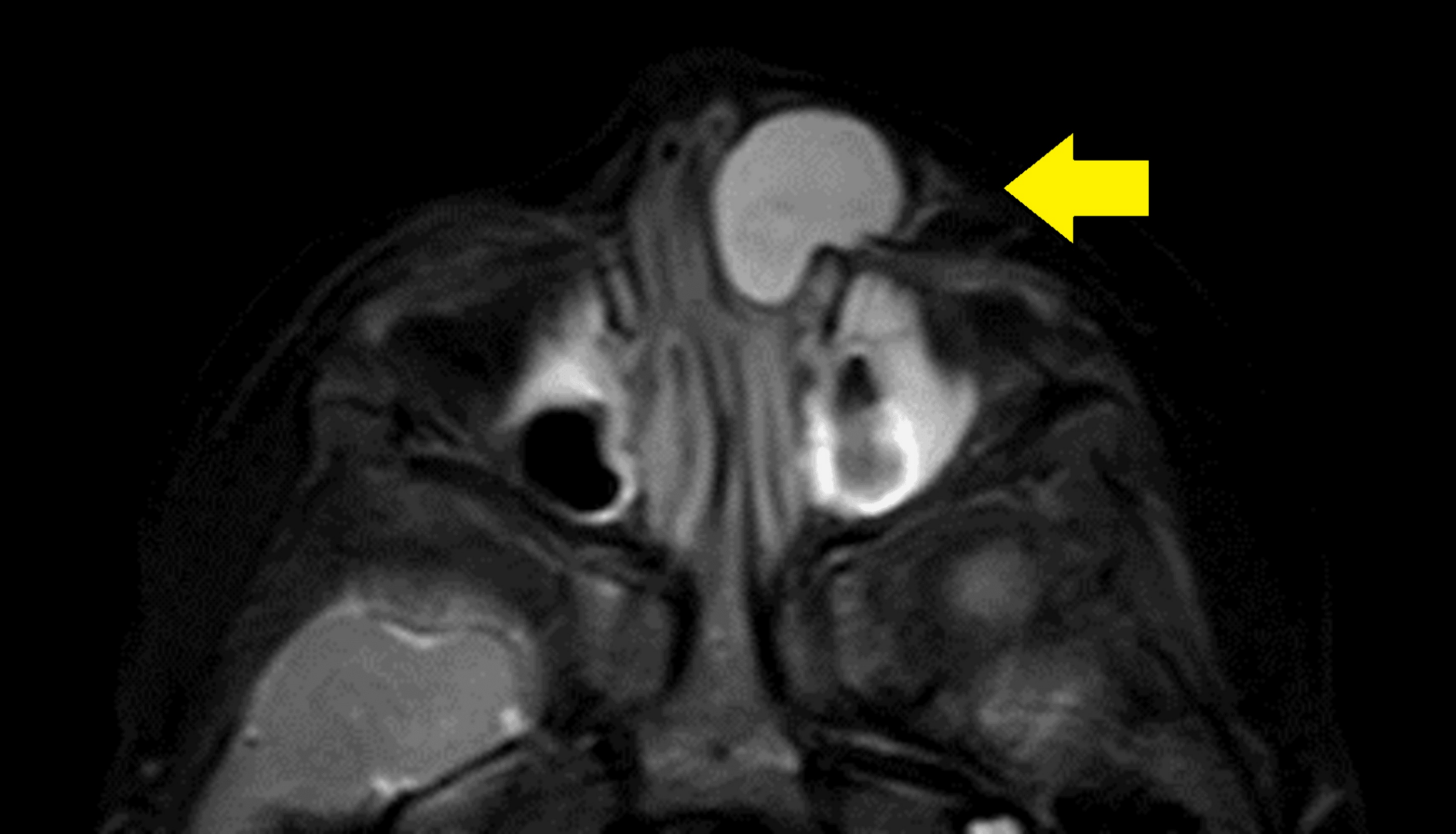
T2-weighted short TI inversion recovery image showing a cystic lesion with high signal intensity.

Upon examination by the plastic surgery department, a tumor was found on the left side of the nasal dorsum measuring 20 mm in size, with no adhesion to the skin (Figure 1a). Plain magnetic resonance imaging (MRI) revealed a cystic lesion with low signal intensity on the T1-weighted image (Figure 2) and high signal intensity on the T2-weighted short TI inversion recovery images (Figure 3). This lesion extended from the left nasolacrimal duct to the anterior wall of the maxillary sinus. The lesion was characterized by well-defined margins and homogeneous internal content. The nasal and maxillary bones exhibited deformation owing to tumor compression. Subsequently, the tumor demonstrated a gradual increase in size. In the three weeks since the first visit, the tumor had grown to 30 mm and was displacing the lower eyelid and nasal dorsum (Figure 1b).

**Figure 4:**
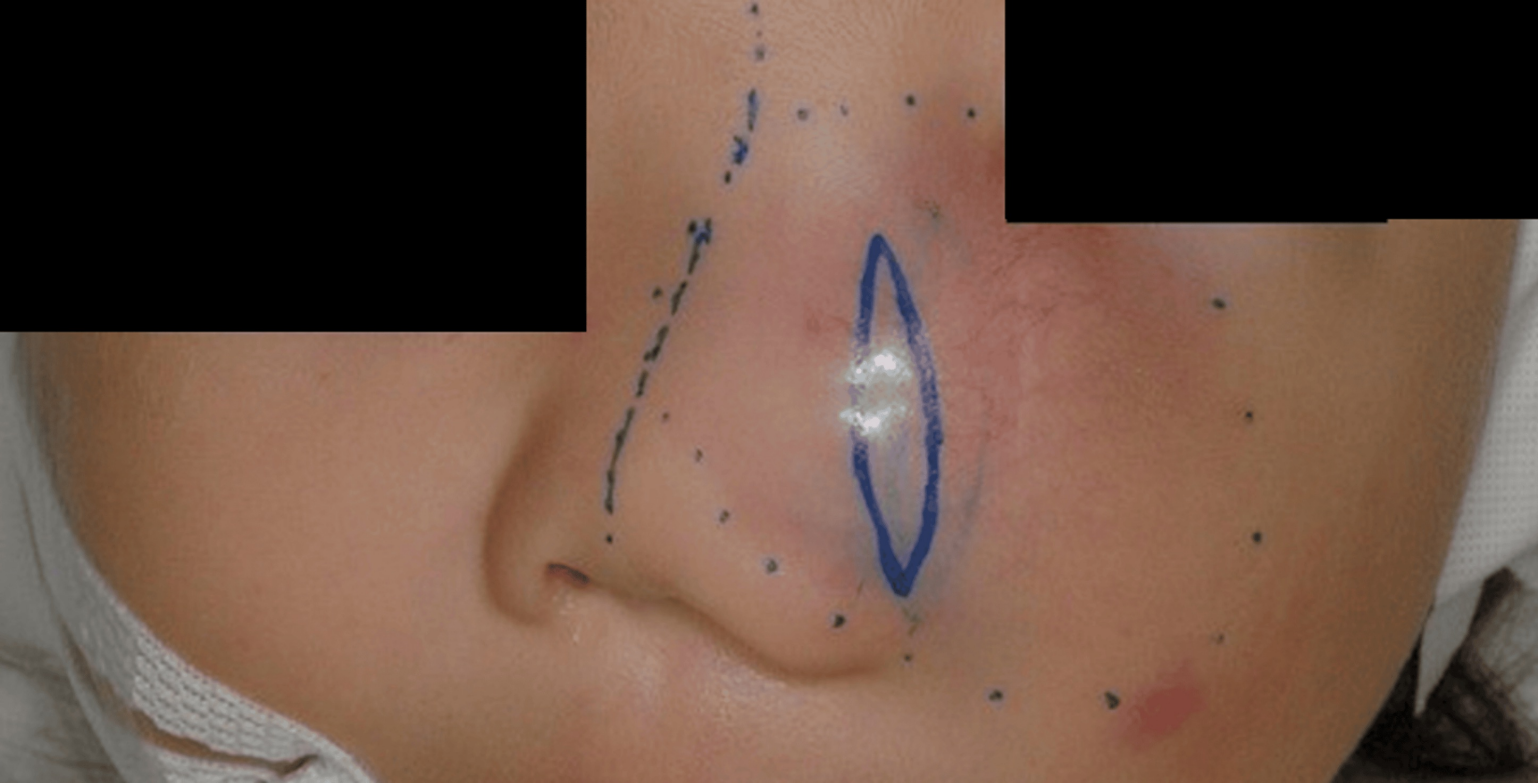
The first surgery was performed through a small incision.

**Figure 5:**
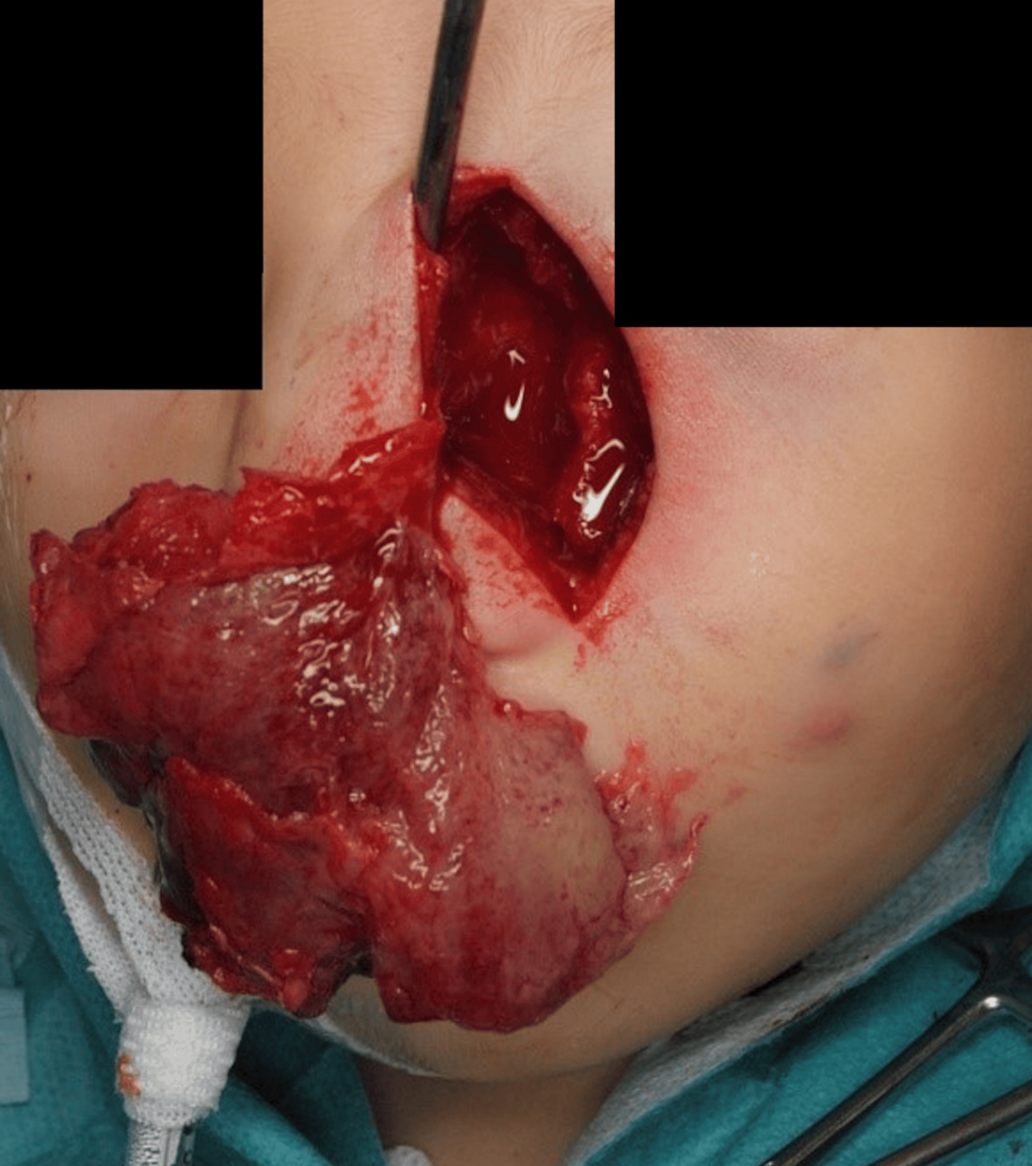
The tumor was resected to the maximum extent possible.

**Figure 6:**
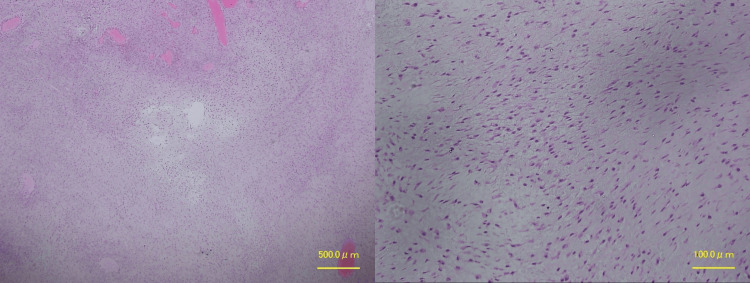
Hematoxylin and eosin staining shows the proliferation of short spindle cells in the stroma of collagen fibers and mucoid substances (×40, ×200)

**Figure 7:**
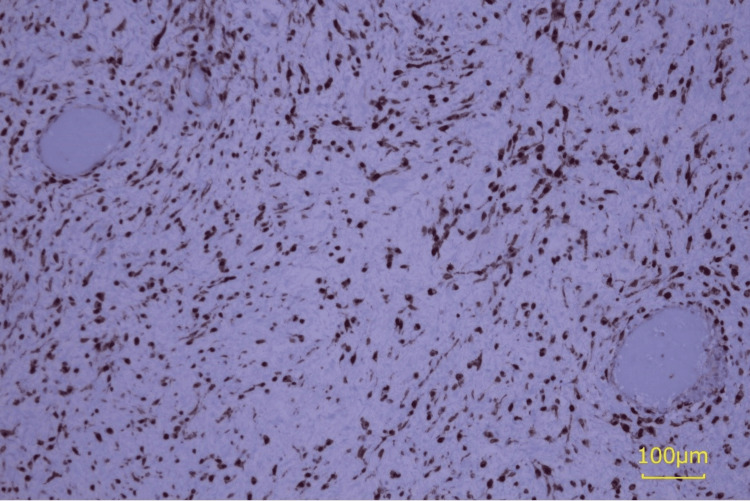
Immunohistochemical staining of β-catenin. β-catenin was strongly positive in the nuclei.

One month after the initial consultation, tumor resection surgery was performed. At that time, the tumor had not definitively been classified as benign or malignant, and an attempt was made to perform as extensive a resection as possible through a small incision (Figures 4, 5). The tumor was an elastic, soft, milky-white solid lesion. The medial infraorbital rim and parts of the nasal bone were excluded by the tumor. Although the tumor could be well-separated from the surrounding soft tissues, it was adherent to the periosteum of the underlying maxilla, necessitating combined resection of the periosteum. The tumor was grossly completely resected as far as could be visualized through a small window. The pathological examination revealed a proliferation of short spindle cells within the stroma of collagen fibers and mucoid substances (Figure 6). Hematoxylin and eosin staining showed no morphological evidence of differentiation into cartilage, fat, or striated muscle. Immunohistochemical tests were negative for S-100, desmin, MyoD1, pan-TRAK, CD34, αSMA, STAT6, SSX, and SS18-SSX; weakly positive for SATB2 and CD99; and positive for vimentin and β-catenin staining. Notably, β-catenin staining was strongly positive in the nuclei of spindle cells (Figure 7). Taking into account the clinical presentation, histopathological features, and growth pattern, the tumor was diagnosed as an SNM.

**Figure 8:**
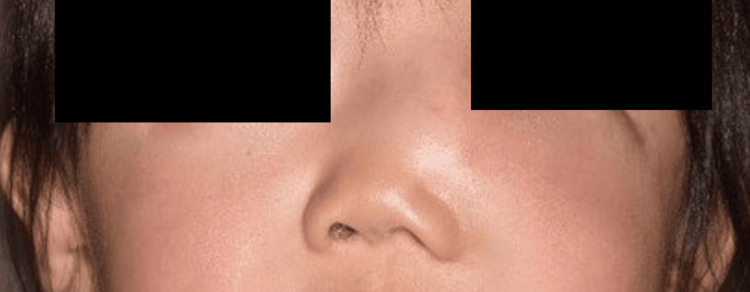
The tumor exhibited signs of recurrence within a few months.

**Figure 9:**
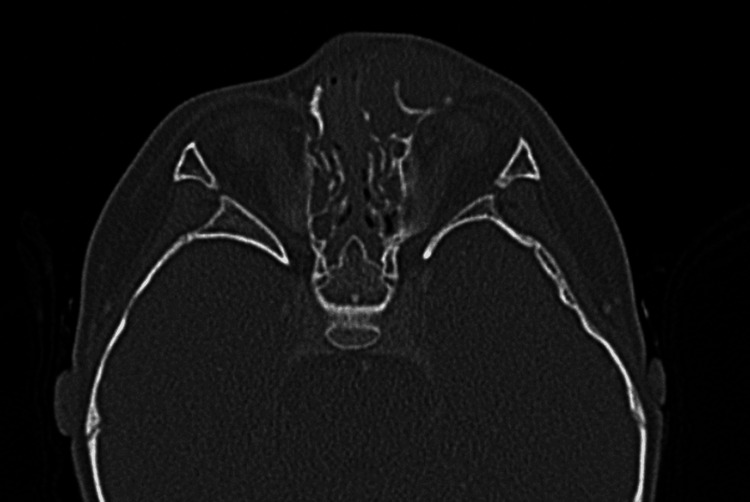
Plain computed tomography showing tumor expansion and bone destruction.

**Figure 10:**
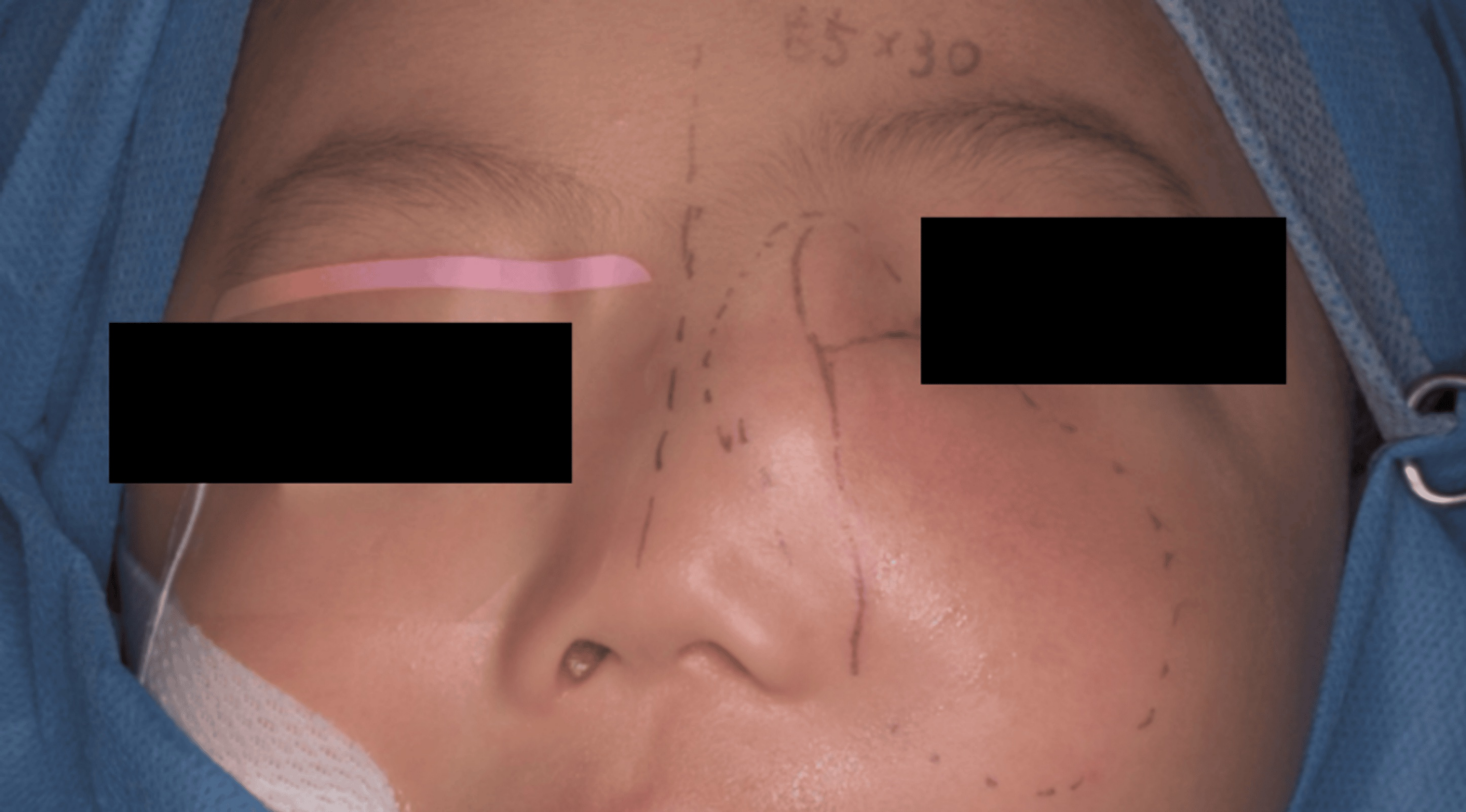
Following the Weber-Ferguson skin incision, a lower eyelid to nasal dorsum incision was performed.

**Figure 11:**
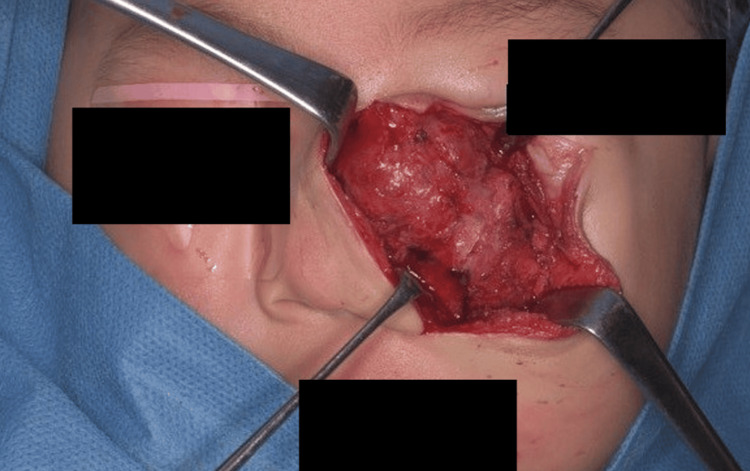
The tumor firmly adhered to the periosteum of the maxilla.

**Figure 12:**
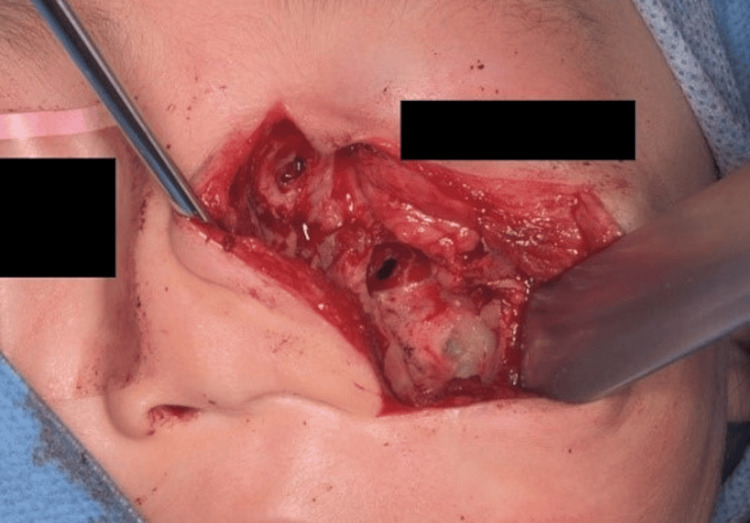
The tumor was then resected. Partial bone resection was performed because the tumor invaded the bone in the inferior orbital rim of the maxilla.

**Figure 13:**
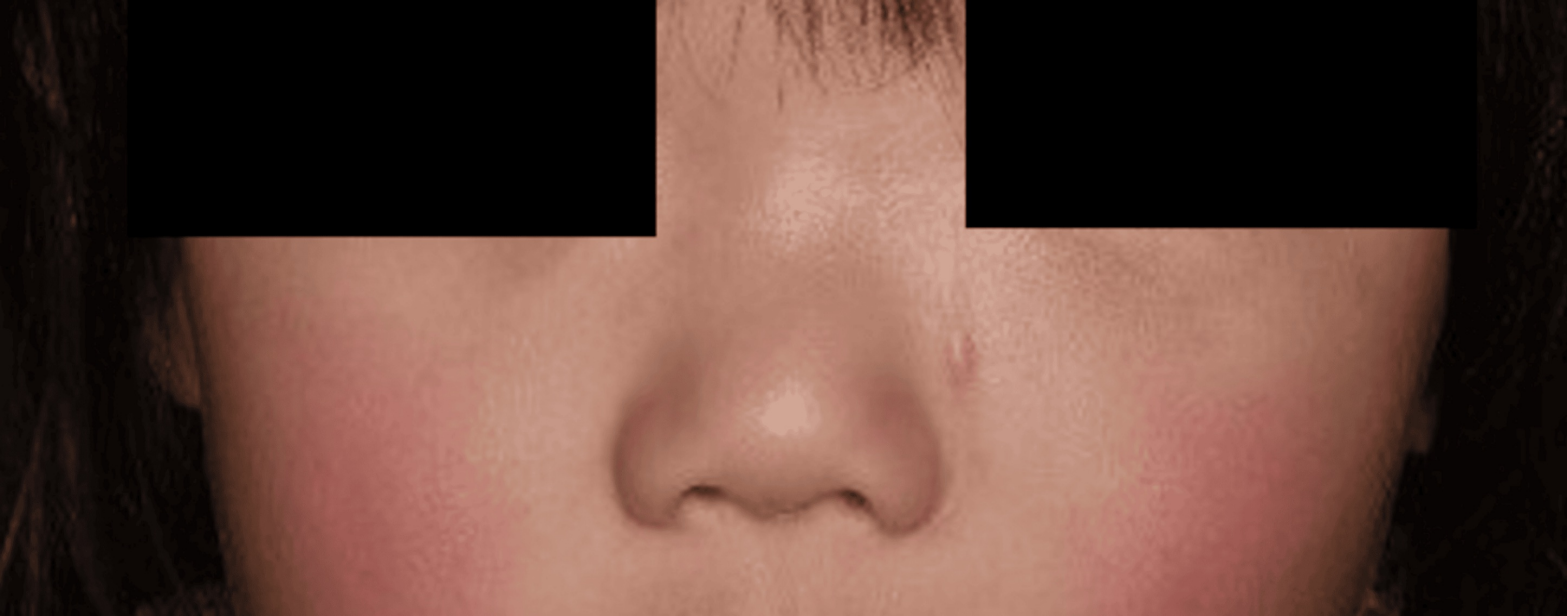
There was no evidence of tumor recurrence at the 12-month follow-up.

Six months after the first operation, the tumor showed signs of recurrence (Figure 8). Although the MRI and computed tomography (CT) images were similar to previous findings, an expansion of the tumor and evidence of bone destruction was observed (Figure 9). Eight months after the initial consultation, the patient was referred to our department for further treatment. The patient underwent a second surgery one month later. Following a Weber-Ferguson skin incision, a lower eyelid-to-nasal dorsum incision was made, and the tumor was grossly resected in its entirety (Figures 10-12). The tumor was easily dissected bluntly from the subcutaneous tissue, albeit with firm adhesion to the periosteum of the maxilla. The periosteum was partially resected. Due to the tumor’s invasion into the bone in the inferior orbital rim of the maxilla, a partial bone resection was also carried out. The medial canthal ligament, lacrimal sac, and nasolacrimal duct were excised because they were involved in the tumor. Reconstruction for the medial canthal ligament was performed by fixing it to the periosteum on the medial side of the orbit. However, reconstruction of the lacrimal sac and nasolacrimal duct was not performed. As a result, the patient is experiencing complications of epiphora. Reconstruction of the lacrimal system is planned after confirming a sufficient period of no recurrence. Pathological examination showed findings similar to those of the previous examination, with complete circumferential interspersion of normal tissues observed around the tumor. No evidence of tumor recurrence was found at the 12-month follow-up (Figure 13).

## Discussion

Myxomas of the head and neck can originate from either soft tissues or the facial skeleton. Those arising from the facial skeleton are commonly found in the mandible and, to a lesser extent, the maxilla. These myxomas are considered to originate from the mesenchymal tissues of the tooth and are referred to as odontogenic myxomas [2,3]. Odontogenic myxomas are more prevalent in females, predominantly in those aged 10-30 years (75%), with a lower occurrence rate in patients younger than 10 years (7.3%) [4,5]. In contrast, for SNMs, which are generally considered non-odontogenic tumors arising from the sinonasal region, the average age of patients is 15.2 months, with a male-to-female ratio of 22:17, indicating no significant sex predilection [6-8]. SNMs are slow-growing, expansile, locally destructive, and infiltrative lesions, with potentially aggressive behavior and the ability to erode bones [9]. To date, no reports have been published on the malignant transformation or distant metastasis of SNM.

There have been 51 reported cases of myxomas occurring in the facial region of patients aged 36 months or younger, of which 49 were confined to the sinonasal complex or maxilla. In addition, 46 of these 49 cases presented clinical features consistent with SNM (Table 1). These lesions, confined to the maxilla, are often considered odontogenic myxomas and are not considered to be separate entities according to the fifth edition of the World Health Organization Classification of Head and Neck Tumours, 2022 [4,10]. However, these cases showed remarkably consistent presentations, including age, location, and other clinical features that diverged from those of odontogenic myxomas. Therefore, myxomas occurring in the sinonasal complex and/or maxilla of infants are suggested to be separate entities from odontogenic myxomas and are referred to as SNMs [5].

**Table 1:** Summary of sinonasal myxomas in children younger than 36 months (1951-2023) SNM: sinonasal myxoma

No.	Year	Author	Age (month)	Sex	Diagnosis	Surgical margin	Recurrence
1	2024	Our case	15	F	SNM	Marginal	+
2	1951	Greenfield and Friedman et al. [11]	24	M	Myxoma of maxillary sinus	Marginal	-
3	1975	Smith et al. [12]	15	F	Odontogenic myxoma	Marginal	+
4	1977	Harris et al. [13]	12	F	Odontogenic myxoma	Marginal	-
5	1977	Fu et al. [14]	15	F	Odontogenic myxoma	Marginal	-
6	1987	James and Lucas [15]	11	F	Odontogenic myxoma	No data	-
7	1990	Leiberman et al. [16]	15	M	Odontogenic myxoma	Marginal	-
	1990	Leiberman et al. [16]	18	F	Odontogenic myxoma	Marginal	-
8	1991	Hayes et al. [17]	12	F	Odontogenic myxoma	Marginal	-
9	1993	Ang et al. [18]	13	M	Odontogenic myxoma	Extended	-
	1993	Ang et al. [18]	15	F	Odontogenic myxoma	Extended	+
10	1993	Caleffi et al. [19]	2	M	Odontogenic myxoma	Marginal	-
11	1993	Heffner [6]	18	M	Odontogenic myxoma	Marginal	-
	1993	Heffner [6]	24	M	Odontogenic myxoma	Marginal	-
12	2000	Brewis et al. [20]	13	M	Odontogenic myxoma	Marginal	-
13	2003	Fenton et al. [21]	17	M	Odontogenic myxoma	Marginal	-
14	2003	Wachter et al. [22]	13	M	Odontogenic myxoma	Marginal	-
	2003	Wachter et al. [22]	19	F	Odontogenic myxoma	Marginal	-
15	2004	Rotenberg et al. [23]	13	F	Odontogenic myxoma	Marginal	-
	2004	Rotenberg et al. [23]	16	F	Odontogenic myxoma	Marginal	-
	2004	Rotenberg et al. [23]	18	M	Odontogenic myxoma	Marginal	-
16	2005	Prasannan et al. [9]	20	F	SNM	Marginal	-
17	2006	Boussault et al. [24]	14	M	Odontogenic myxoma	Marginal	-
18	2008	King et al. [25]	17	M	Odontogenic myxoma	Marginal	-
	2008	King et al. [25]	18	M	Odontogenic myxoma	Marginal	-
19	2010	Iatrou et al. [7]	12	M	SNM	Marginal	-
20	2011	Safadi et al. [3]	20	M	SNM	Marginal+partial maxillary resection	-
21	2012	Kansy et al. [26]	11	No data	Odontogenic myxoma	Marginal+partial maxillary resection	+
	2012	Kansy et al. [26]	12	No data	Odontogenic myxoma	Marginal	-
22	2012	Ríos Y Valles-Valles et al. [27]	11	F	Odontogenic myxoma	Marginal	-
23	2013	Kadlub et al. [28]	14	F	Odontogenic myxoma	Marginal	+
	2013	Kadlub et al. [28]	18	F	Odontogenic myxoma	Marginal	-
	2013	Kadlub et al. [28]	21	F	Odontogenic myxoma	Marginal	+
	2013	Kadlub et al. [28]	23	M	Odontogenic myxoma	Marginal	-
24	2013	Zainine et al. [29]	2	No data	Odontogenic myxoma	Marginal	-
25	2013	Chen et al. [30]	15	F	Odontogenic myxoma	Marginal	-
26	2016	Subramaniam et al. [31]	24	No data	Odontogenic myxoma	Extended	-
27	2016	Kelly et al. [32]	5	M	SNM	Marginal	-
28	2016	Hansen et al. [33]	36	M	Odontogenic myxoma	Marginal+partial maxillary resection	-
29	2020	Mewar et al. [1]	11	M	SNM	Marginal	-
30	2021	Kondamuri et al. [34]	9	M	SNM	Marginal	-
31	2023	Velez Torres et al. [35]	20	M	SNM	Marginal	+
	2023	Velez Torres et al. [35]	25	M	SNM	Marginal	No data
	2023	Velez Torres et al. [35]	25	M	SNM	Marginal	No data
	2023	Velez Torres et al. [35]	25	F	SNM	Marginal	No data
	2023	Velez Torres et al. [35]	36	F	SNM	Marginal	-
			Average: 15.5M	M: 23, F: 19			

Typical clinical symptoms of SNM include painless swelling in the nasolabial or maxillary sinus regions. Other common symptoms include epistaxis and nasal obstruction, and if the tumor extends into adjacent structures, pain, malocclusion, or diplopia may occur.

Typically, on radiographic imaging, SNMs show well-defined, expansile, unilocular, or multilocular radiolucent lesions. CT and MRI show well-bordered cystic lesions and often show bone compression and infiltration into the bone [1,3]. Grossly, myxomas appear as grey-white, nodular, shiny masses with a gelatinous or mucoid appearance that lack a true capsule [3].

Histologically, odontogenic myxomas and SNMs are similar, consisting of non-encapsulated, loosely proliferating spindle, stellate, or round cells with small hyperchromatic nuclei and thin cytoplasmic projections in a fibromyxoid to mucinous background stroma. Pleomorphism, mitotic figures, and necrosis are usually absent.

In some cases of odontogenic myxomas, odontogenic epithelia are found within the tumor, supporting an odontogenic origin [10]. However, SNMs have not been associated with odontogenic epithelia in all previous reports including which is reported as odontogenic myxoma. Immunohistochemistry has indicated that SNMs exhibit characteristics similar to those of odontogenic myxomas in many aspects. However, while odontogenic myxomas are generally negative for β-catenin staining, positive staining has been reported in SNMs [1,26,35]. In the present case, the cell nuclei were positive for β-catenin staining, which was used as a diagnostic aid. Furthermore, SNMs share genetic mutations with desmoid tumors, which are not observed in odontogenic myxomas [35]. These findings have led to the proposition that SNMs are distinct entities, separate from odontogenic myxomas, and may potentially be classified under the desmoid tumor category, as infantile intraosseous myxoid desmoid fibromatosis [35].

No previous reports have concurrently investigated β-catenin immunostaining and genetic mutations in SNMs. Though further studies are needed, the findings of the present case report, coupled with the characteristic patient profiles, clinical presentations, and pathological features observed in numerous previous reports on SNMs, suggest that SNM may indeed be a distinct tumor, separate from odontogenic myxoma, possibly belonging to a different category.

The differential diagnoses of SNM based on the clinical, radiographic, and histological findings include fibrous dysplasia, odontogenic cysts, benign tumors, and pediatric sarcomas (especially rhabdomyosarcoma). A careful interpretation of biopsy specimens is crucial, as the predominant mucinous components in tissue morphology have led to initial misdiagnoses such as fibrous dysplasia, nodular fasciitis, infantile fibromatosis, and rhabdomyosarcoma. These were later correctly diagnosed as SNMs or odontogenic myxomas (clinically considered SNM).

The standard treatment protocol for SNMs in infants generally involves surgical excision of the tumor. However, owing to the limited number of cases, no systematic reports are available on surgical approaches or margins. In the context of odontogenic myxomas, recurrence rates of 31.3% with curettage, 13.1% with enucleation, and 1.3-6.7% with excision involving bone resection have been reported [36]. Some reports advocate for extended excision with ample margins, whereas others recommend marginal excision to preserve the surrounding tissues [23]. However, in previous SNM cases, 42 of 45 cases (93.3%) underwent marginal excision with or without partial bone resection around the area of bone destruction. Among those cases that underwent marginal excision, recurrence was observed in six cases (14%) (Table 1). Considering that the tumor in the present case occurred in the facial region, marginal excision was deemed preferable. To date, no reports have indicated that SNMs respond to radiation or chemotherapy. However, advancements in the histological classification of SNM may prompt the consideration of chemotherapy in the future. In the present case, the first surgery did not achieve complete macroscopic and histological excisions, leading to recurrence. However, the second surgery involved a complete excision with no visible residual tumor, and no recurrence has been reported to date.

## Conclusions

We presented a case of SNM in a 15-month-old infant. The patient experienced recurrence after the first surgery. However, following marginal excision during the second surgery, no recurrence has been reported to date. Given its consistent clinical features and its distinct histological, immunohistochemical, and genetic profiles compared to odontogenic myxomas, SNM may be classified as a separate disease entity.
